# Promoting Late-Life Volunteering With Timebanking: A Quasi-Experimental Mixed-Methods Study in Hong Kong

**DOI:** 10.1093/geroni/igae056

**Published:** 2024-06-04

**Authors:** Shiyu Lu, Cheryl Chui, Terry Lum, Tianyin Liu, Gloria Wong, Wai Chan

**Affiliations:** Department of Social and Behavioural Sciences, City University of Hong Kong, Kowloon, Hong Kong, China; Department of Social Work and Social Administration, The University of Hong Kong, Pok Fu Lam, Hong Kong, China; Department of Social Work and Social Administration, The University of Hong Kong, Pok Fu Lam, Hong Kong, China; Department of Social Work and Social Administration, The University of Hong Kong, Pok Fu Lam, Hong Kong, China; Department of Social Work and Social Administration, The University of Hong Kong, Pok Fu Lam, Hong Kong, China; School of Psychology and Clinical Language Sciences, University of Reading, Reading, UK; Sau Po Centre on Ageing, The University of Hong Kong, Pok Fu Lam, Hong Kong, China

**Keywords:** Active aging, Productive aging, Volunteerism

## Abstract

**Background and Objectives:**

This study explores the impact of timebanking, where individuals earn time credits, nonmonetary currency, on promoting volunteerism among older adults.

**Research Design and Methods:**

This study employed a quasi-experimental design with 116 timebank group (TBG) participants and 114 comparison group (CG) participants from 2021 to 2022. TBG received time credits to exchange for rewards, while CG received no time credits (i.e., volunteering as usual). The intervention of timebanking lasted for 1 year. Volunteering behaviors were tracked via an app, and intentions to volunteer were assessed at baseline (T0), after 6 months (the midpoint of the intervention, T1), and after 12 months (the endpoint of the intervention, T2). The use of rewards by TBG participants was also recorded (e.g., for personal use or sharing with others). Furthermore, focus group interviews were conducted to understand how rewards influenced participants’ volunteerism.

**Results:**

TBG had significantly higher weekly volunteer hours at T2 (β = 1.37, *p* = .021) and increased intent to volunteer at T1 (β = 0.54, *p* = .001) and T2 (β = 0.51, *p* = .001) compared with CG. Participants using rewards personally volunteered more at T2 (β = 2.09, *p* = .014), although sharing rewards with family and friends or donating rewards to others did not yield the same effect. The qualitative study suggested that a sense of feeling recognized generated by timebanking may encourage increased volunteering and that personal reward use enriched the volunteer experience, and individuals sharing rewards with family and friends experienced a sense of fulfillment and reinforcement of their prosociality.

**Discussion and Implications:**

Timebanking effectively encourages late-life volunteering. The study provides practical implications for promoting volunteering among older people.

**Translational Significance:** Timebanking is a promising initiative to promote late-life volunteering, although it lacks strong empirical support. This quasi-experimental mixed-method study addresses this gap, demonstrating that reward-based timebanking significantly enhances volunteer hours and intentions compared with the nonreward condition. This qualitative analysis further revealed that people who used rewards from timbanking personally experienced an enriched volunteering experience, and those who shared rewards with family and friends experienced a sense of fulfillment and reinforcement of their prosociality. Social service organizations and governments should consider timebanking as an effective tool to promote late-life volunteering.

The rising demand for health and social care driven by rapid population aging and care workforce shrinkage requires innovative initiatives to harness the time and energy of older adults (Organization for Economic Cooperation and Development, 2023). Many older people in their 60s and 70s remain capable of contributing to society (Morrow-Howell & Mui, 2014). This stance echoes policy efforts over recent decades that encourage productive engagement in later life. Productive engagement encompasses unpaid and paid activities that generate goods and services for society, such as volunteering (Hinterlong et al., 2003). Late-life volunteering has gained increasing attention in health promotion (Filges et al., 2020), as volunteering contributes to better health, prevention of depression, and increased quality of life among older adults (Chan et al., 2021; Filges et al., 2020; Lum & Lightfoot, 2005; Morrow-Howell et al., 2003). Promoting volunteering can also facilitate mutual help and strengthen community caring capacity, potentially reducing reliance on care professionals (Fernandes-Jesus et al., 2021; Lu et al., 2022). Hence, identifying effective strategies to promote large-scale volunteering in later life is imperative.

One potential innovative strategy is to incorporate timebanking into the social care system. Edgar Cahn initially conceived of timebanking in the 1980s as a platform to facilitate service exchanges (e.g., peer support, escorts) using time as an alternative currency (Cahn, 2000; Collom, 2008). Each hour a member contributes to their community through volunteering activities is rewarded with a time credit (Cahn & Gray, 2021). The earned time credits can be exchanged either for another person’s time, services (e.g., cleaning or grocery shopping), or rewards provided by an organization (e.g., day trip; Collom, 2007). Unlike traditional volunteering, timebanking offers volunteers the autonomy to choose their rewards. Volunteers can decide the type and quantity of their rewards based on accumulated time credits, making the experience more personalized and fulfilling. Preliminary evidence suggests that timebanking encourages volunteerism among members (Bretherton & Pleace, 2014; Collom, 2008) and is recommended by the World Health Organization (2015) as a strategy to foster late-life volunteering. However, robust evidence supporting the effects of timebanking on promoting later-life volunteering is lacking. Lee et al. (2020) systematic review revealed a scarcity of high-quality research studies on the effectiveness of timebanking on volunteerism. Most studies were small-scale case studies or local evaluations (Kimmel, 2015; Sanz, 2016), and others adopted qualitative methods (Seyfang, 2005). Thus, robust evidence concerning the effectiveness of timebanking in promoting later-life volunteering is needed.

Social exchange theory (SET) provides insights into timebanking’s potential to promote volunteerism. SET posits that social exchanges are grounded in cost-benefit analyses (Gouldner, 1960; Wahrendorf et al., 2010), emphasizing reciprocity as the principle that individuals participating in social relationships should receive equitable rewards (Gouldner, 1960). When individuals perceive that the benefits they receive are less than their contributions, they are more likely to experience stress and negative emotions, leading to the possible termination of these relationships (Sakman, 2019). This cost-benefit analysis is not limited to dyadic social exchange. It also extends to situations where social exchange activities, like volunteering, yield benefits for a broader range of individuals and organizations (Cropanzano & Mitchell, 2005). Volunteering can be viewed as a costly social exchange activity as it often demands investing personal time and energy in the greater social good (Tse, 2020). Therefore, rewards may offset the costs assumed by volunteers. Emerging observational studies have found that older volunteers who feel rewarded and recognized report more positive experiences, reinforcing their volunteer engagement (Tse, 2020; Zaninotto et al., 2013). Timebanking offers a social exchange mechanism allowing older people to earn time credits from volunteering and operates on the premise that one hour of volunteering equates to one time credit that can be exchanged for preferred rewards and services (Cahn & Gray, 2021). Based on SET, timebanking could promote volunteering among older adults.

Furthermore, there is a noticeable lack of evidence about the effect of reward-sharing behaviors among volunteers on subsequent volunteer engagement and intentions. Social discounting theory (SDT) suggests that an individual’s perceived social discounting rates on the value of rewards vary according to the type of relationship with the people with whom the reward would be shared (Ostaszewski & Osiński, 2011). Ostaszewski and Osiński (2011) found that social discounting of monetary rewards is most pronounced when the rewards were shared with strangers and least/modest when shared within the volunteers’ social circles, such as family or friends. Although older adults are regarded as being more generative towards others, Gong et al. (2019) revealed that older adults have been shown to prefer socially proximate individuals over distant others when making monetary donations. A qualitative study found that older volunteers may be more engaged in volunteering if the rewards benefitted them directly or were shared with relatives or friends (Lu et al., 2023). This inclination can be attributed to their perception of rewards as personal gains offsetting the costs of volunteering when they keep rewards for themselves or share rewards with family and friends. Conversely, the act of donating rewards to strangers may not produce a similar effect. Examining how the type of relationship with people with whom the rewards are shared shapes volunteers’ subsequent volunteerism could enhance our understanding of the reward design, further facilitating the development of strategies to sustain late-life volunteering (Gong et al., 2019).

## The Current Study

This study explored the impact of timebanking on promoting late-life volunteering. Consistent with SET, we hypothesized that timebanking would effectively foster volunteer participation (Hypothesis 1.1), volunteer hours (Hypothesis 1.2), and volunteer intentions (Hypothesis 1.3) compared with a nonreward condition. Additionally, guided by SET and SDT, we hypothesized that volunteers retaining the rewards for themselves (Hypothesis 2.1) and distributing rewards within their social circles (Hypothesis 2.2) would positively influence subsequent volunteerism. To gain a deeper understanding of these dynamics, we also implemented a qualitative approach to explore participants’ perceptions of rewards and their experiences when utilizing these rewards, shedding light on the mechanisms through which timebanking influences late-life volunteerism.

## Method

### Study Design

The study used a quasi-experimental mixed-method design in Hong Kong to evaluate the effectiveness of timebanking in promoting late-life volunteering. We initially classified three districts (Eastern, Southern, and Wong Tai Sin) as reward districts and three other districts (Central & Western, Wan Chai, and Kwun Tong) as comparison districts, where participants did not bank their time credits or receive rewards. Based on the 2021 Population Census (Hong Kong Census and Statistics Department, 2021), the chosen districts for both the timebank and comparison groups were comparable in terms of their socioeconomic situations (see Supplementary Table 1). Subsequently, we conducted qualitative focus group interviews as a supplementary method after timebanking implementation, allowing participants to discuss their perceptions of rewards, experiences using rewards, and the influence of rewards on their volunteering. We registered this study with the Centre for Open Science (https://osf.io/dmfvh). The Research Ethics Committees of the University of Hong Kong and City University of Hong Kong approved the study.

### Quasi-Experimental Study Design

#### Participant recruitment

**Figure 1.**
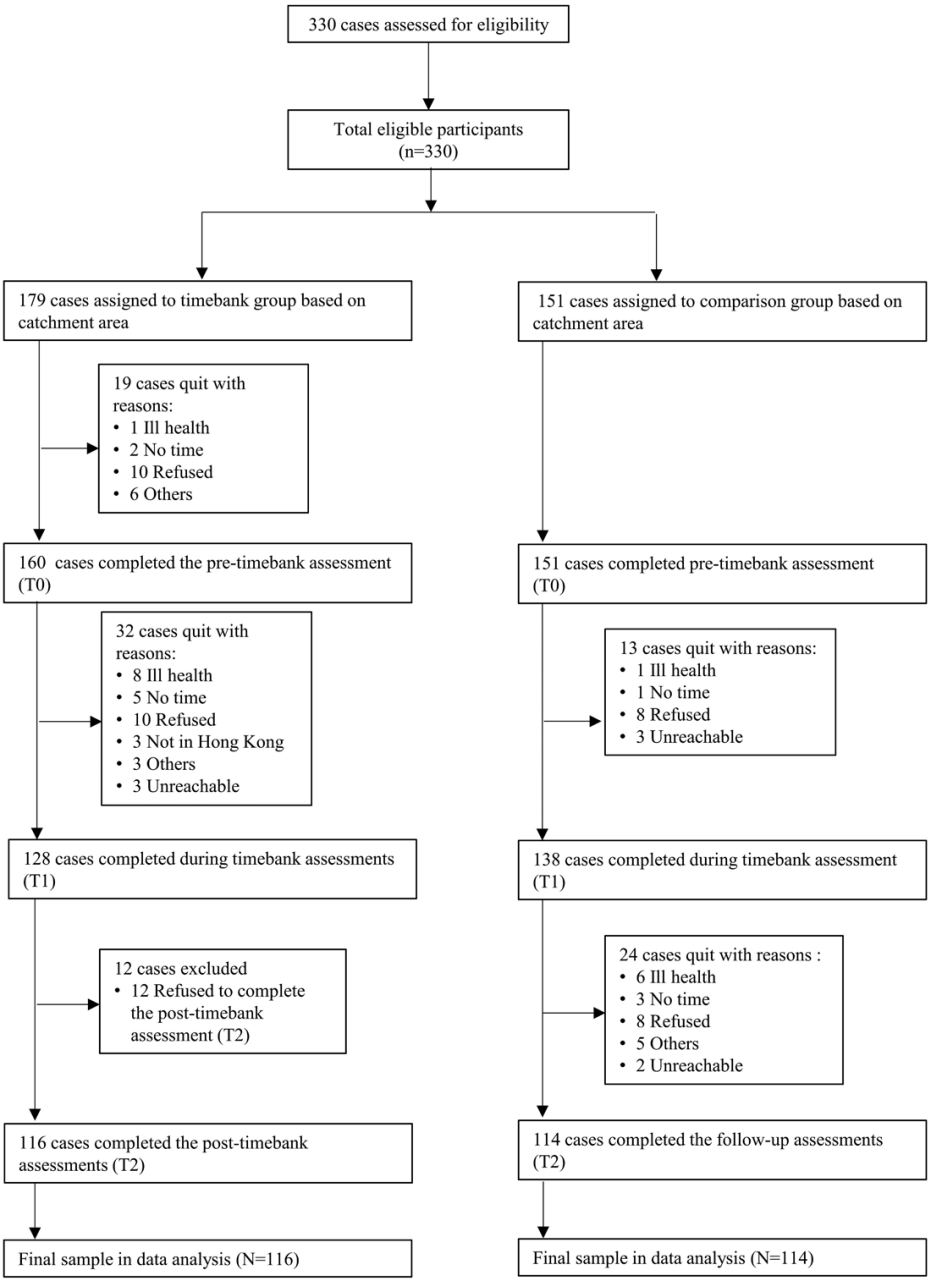
Flow chart.

The eligibility criteria for both timebank and comparison groups included being 50 years or older, fluent in Cantonese, understanding written Chinese, and residing/serving in the target areas (e.g., timebank districts vs comparison districts). The age criteria aligned with prior longitudinal studies related to volunteering (Kim et al., 2020) and was relevant for individuals transitioning to retirement. We collaborated with 21 community centers for older adults to recruit participants for the timebank and comparison groups under the project, “Health Ambassador Program” (HA Program hereafter). Between January 2021 and February 2022, we invited 330 older individuals through flyer distribution. In the timebank group, we recruited 179 participants, and 160 completed the pretimebank assessment. In the comparison group, we recruited 151 participants, all of whom completed the pretimebank assessment. At the follow-up stage, 116 participants in the timebank group and 114 in the comparison group completed the surveys. The dropout rates were 35.1% and 24.5% for the timebank and comparison groups, respectively (see Figure 1).

#### Data collection

Data were collected at 3 time points for both groups: at baseline (T0, February 2021), after 6 months (the midpoint of the intervention, T1), and after 12 months (the endpoint of the intervention, T2). The T0 surveys were conducted face-to-face. However, due to the impact of COVID-19, follow-up surveys were carried out through an online self-administered mode, with the option of a telephone survey for participants requiring assistance. A trained research assistant managed the data collection process.

#### The settings of timebanking and the stimulation procedures

The timebanking setup consisted of three components: volunteer opportunities, a volunteer hours record platform, and the exchange of time credits for rewards. The first two components were the same for both the timebank and comparison groups, and the third was exclusive to the timebank group.

First, after the T0 survey, participants from both groups were assigned to the community centers to engage in various volunteering activities under the HA program, such as assisting in workshops and making home visits or phone calls to service recipients.

Second, all participants in both groups were introduced to an online platform, “HINCare App” (HINCare), operated by the Department of Computer Science at The University of Hong Kong. HINCare is a mobile app that allows participants to sign up for volunteering opportunities and log their hours. Both groups received training on using HINCare, and all partner community centers had administrative accounts to post volunteer opportunities.

Lastly, only the timebank group was informed that their volunteer hours would be converted into time credits in HINCare. We created an “Exchanging for Rewards” section in the app exclusively for the timebank group. Participants could use their time credits to obtain various rewards (e.g., a day trip, home-made cookies, and dining coupons in a social enterprise restaurant) from partner NGOs and social enterprises. A list of rewards, exchange rates, and the social values of reward providers were displayed in the “Exchanging for Rewards” section. The exchange rate was set at HK$20 per time credit based on feedback from a 2019 survey of 130 older volunteers; this rate was then used to determine the value of rewards. Due to COVID-19 restrictions, rewards became available 6 months after the announcement, making the total duration of timebanking intervention 12 months. A trained research assistant managed the reward exchange process. Before officially launching the reward section, we piloted it with the assistance of several older adult volunteers. The comparison group did not bank their time credits or receive any rewards.

#### Rewarding sharing: Self-use and close social circles versus rewarding sharing to strangers

In the HINCare App, we implemented a reward selection system for the timebank group. When claiming rewards using time credits, participants were presented with three options to indicate their reward-sharing intentions: “self-use,” “sharing with family/friends,” or “donating to others,” with the possibility of selecting more than one option. Next, we recoded the responses for each category. For instance, for the “self-use” category, if a participant chose “self-use” at least once, the response was coded as “1.” If this option was not selected, it was coded as “0.” Similar recoding processes were applied to the “rewards sharing with family/friends” and “donating to others” categories.

#### Outcome measures

The outcomes included volunteer behaviors and intentions, specifically, participation, hours, and intention to volunteer. *Volunteer participation* was evaluated using two sources: (1) self-reported participation in formal voluntary work beyond the HA program at 3 time points over the past 6 months (1 = Yes, 0 = No); and (2) participation in voluntary work within the HA program, as tracked by the HINCare. Participants were coded as “participated” (1) if they engaged in voluntary work within or beyond the HA program, or “not participated” (0) otherwise. *Weekly volunteer hours* were calculated using two sources: (1) self-reported volunteer hours beyond the HA program at three time points, with participants indicating their weekly volunteer hours over the past 6 months; and (2) volunteer hours within the HA program, as recorded by the HINCare. We calculated weekly volunteer hours for each time point by summing the hours within and beyond the HA program and converting the sum into weekly hours based on the number of weeks in the periods. Capturing the volunteer behaviors beyond and within the HA program provided a comprehensive understanding of volunteerism among older people. To measure *volunteer intention*, participants were asked a single question about the likelihood of their volunteering in the coming 3 months (a 5-point Likert scale: 1 = very unlikely, 5 = highly likely).

#### Covariates

Participants’ sociodemographic characteristics were collected, including gender (male/female), age, education (primary and below/secondary/postsecondary), marital status (married/others), monthly income levels, employment status, number of years of volunteering experience, number of years resident in their districts, their participation in different types of voluntary work (e.g., social service, religious, regional organizations, and others) in the past 12 months and self-rated health (poor, fair or good) at T0. Given the impact of COVID-19 on volunteer opportunities, participants were also asked to rate their perception of whether such opportunities decreased during the pandemic on a scale of 1 (strongly disagree) to 5 (strongly agree).

### Data Analysis

We conducted descriptive statistics for demographics, perceived volunteer opportunities during the pandemic, and outcome variables at baseline. Independent samples *t*-tests were used for mean comparisons of continuous variables, and chi-square tests for categorical variables between the timebank and comparison groups. To test Hypotheses 1.1–1.3, mixed models with random intercepts controlling for covariates were applied to account for observations nested within participants across three time points. This method is superior to traditional repeated-measures analysis as it uses all available data per subject and is impervious to random missing data (Gueorguieva & Krystal, 2004). For Hypothesis 1.1, we utilized logistic mixed models with interaction terms (groups × time points) to gauge the adjusted differences in volunteer participation between the timebank and comparison groups from T0 to T2, as recommended by Hilbert et al. (2019). Hypothesis 1.2 was tested by employing a generalized linear mixed model with interaction terms to estimate the adjusted mean differences (AMDs) in volunteer hours, as some participants did not volunteer, thus skewing the distribution of volunteer hours. For Hypothesis 1.3, a linear mixed model with interaction terms was used to calculate AMDs in volunteer intentions. Hypothesis 4 was tested using a generalized linear model (GLM) and linear regression controlling for covariates to investigate the impact of rewards for self-use, sharing with relatives and family, and donating to strangers on volunteer hours and intentions at T2. The skewed distribution of volunteer hours necessitated the use of GLM. The rewards exchanges were only available 6 months after announcing the timebank (between T1 and T2), so reward-sharing behaviors only influenced volunteer hours and intentions at T2. As covariate variables had <5% missing rate, multiple imputations by chained equations were performed to handle missing data, which were missing completely at random based on Little’s test (Li, 2013). All the data analyses were conducted using STATA 15.

### Qualitative Study

We conducted semi-structured focus group interviews to better understand participants’ perceptions of rewards, their experiences using rewards, and the impact of using rewards on volunteering, supplementing the findings from the quasi-experimental study. We used purposeful sampling to recruit participants from the timebank group based on two conditions: (1) not claiming rewards and (2) claiming rewards. We invited participants from each condition to join three separate focus groups, each comprising around six participants—17 participants in total. Supplementary Table 2 provides participant details.

After obtaining informed consent, we conducted semistructured interviews eliciting participants’ experiences of their engagement in the timebanking. Sample questions included “What is your perception of the rewards in the timebank project?” “Please share your experience using the rewards claimed via time credits under the timebank project,” “Did the presence of rewards shape your volunteer hours or intention? If so, why, and how? If not, why not?’ We also explored reasons for not claiming rewards. We conducted all three focus groups at the university campus between November and December 2022. The interviews were audiotaped and transcribed verbatim. The number of focus groups was decided by data saturation. The first author moderated all the focus group sessions, and a research assistant took notes. Each focus group lasted about 90 min. All focus groups were conducted in Cantonese, participants’ first language. Illustrative quotations used in this article have been translated into English. We conducted thematic analysis to develop, analyze, and interpret patterns across the data collected from the focus groups (Clarke & Braun, 2021). Thematic analysis involves an active process of reflexivity (Braun & Clarke, 2019; Clarke & Braun, 2021). Following Clarke and Braun (2021) six phases, we familiarized ourselves with the data, generated an initial list of codes, grouped them into emergent themes, refined them, and defined/named them for reporting. Two independent researchers repeated the first three stages for reflexivity and a nuanced understanding of the data. We integrated quantitative and qualitative findings to illustrate how rewards and their usage shaped volunteer behaviors.

## Results

### Findings of the Quasi-Experimental Study

Table 1 displays the demographics and baseline characteristics for the timebank and comparison groups. The mean age of participants was 64.83 (*SD* = 6.16); most were female (80.9%), married (61.3%), completed their education at secondary level or below (65.7%), and had a monthly income below HK$6 000 (around US$770) (66.1%). The average length of residence in their districts was 31.35 years (*SD* = 18.51), and their volunteering experience was 9.36 years (*SD* = 10.59). Around half volunteered in social service-related activities in the past 12 months at T0. The groups were comparable in gender, marital status, economic status, self-rated health, length of residence, and perceived volunteering opportunities during the pandemic. Two differences were observed: the timebank group was younger (Mean = 63.49, *SD* = 5.6) than the comparison group (*M* = 66.2, *SD* = 6.43) (*t* = 3.41, *p* < .001), and the comparison group had more volunteering experience (*M* = 10.77, *SD* = 11.8) than the timebank group (*M* = 7.92, *SD* = 9.02) (*t* = 2.01, *p* < .05).

**Table 1.** Sample Characteristics and Group Comparison (*N* = 230)

Variables	Total (*N* = 230)	Timebank group (*n* = 116)	Comparison group (*n* = 114)	Chi-square/*t*-test
	*N* (%) / Mean (*SD*)	*N* (%) / Mean (*SD*)	*N* (%) / Mean (*SD*)	
Gender				0.004
Male	44 (19.1%)	22 (19.0%)	22 (19.3%)	
Female	186 (80.9%)	94 (81.0%)	92 (80.7%)	
Age in years	64.83 (6.16)	63.49 (5.60)	66.2 (6.43)	3.41***
Age group (range = 52–89)				10.15**
50–59 years	46 (20.0%)	28 (24.1%)	18 (15.8%)	
60–69 years	133 (57.8%)	72 (62.1%)	61 (53.5%)	
70 and above	51 (22.2%)	16 (13.8%)	35 (30.7%)	
Marital status				1.108
Married	141 (61.3%)	75 (64.7%)	66 (57.9%)	
Single/widowed/divorced/separated/others	89 (38.7%)	41 (35.3%)	48 (42.1%)	
Education				5.223
Primary or below	25 (10.9%)	14 (12.1%)	11 (9.6%)	
Secondary	126 (54.8%)	55 (47.4%)	71 (62.3%)	
Tertiary or above	79 (34.3%)	47 (40.5%)	32 (28.1%)	
Monthly salary (HK$)				4.167
Below $2,000	111 (48.3%)	55 (47.4%)	56 (49.1%)	
$2,000–$5,999	41 (17.8%)	17 (14.7%)	24 (21.1%)	
$6,000–$9,999	22 (9.6%)	15 (12.9%)	7 (6.1%)	
$10,000 and above	56 (24.3%)	29 (25.0%)	27 (23.7%)	
Employment status				
Employed/self-employed	23 (10.0%)	14 (12.1%)	9 (7.9%)	1.19
Retired/homemaker/unemployed	201 (87.4%)	96 (82.8%)	105 (92.1%)	
Self-rated health				1.43
Good health	159 (69.1%)	81 (69.8%)	78 (68.4%)	
Poor or fair health	69 (30%)	35 (30.2%)	34 (29.8%)	
Length of residence in their districts (range = 1–76 years)	31.35 (18.51)	30.67 (18.68)	32 (18.41)	0.533
Length of volunteering experience (range = 1–50 years)	9.36 (10.59)	7.92 (9.02)	10.77 (11.80)	2.01*
Types of volunteering work in the past 12 months				
Social service-related	117 (56.3%)	58 (58.0%)	59 (54.6%)	0.24
Religious-related	35 (16.8%)	16 (16.0%)	19 (17.6%)	0.09
Regional organization	32 (15.4%)	14 (14.0%)	18 (16.7%)	0.28
Others	54 (26.0%)	31 (14.5%)	23 (21.3%)	2.54
None	32 (15.4%)	12 (12%)	20 (18.5%)	1.69
Volunteering participation rate in the past 6 months at baseline	132 (57.4%)	76 (65.5%)	56 (49.1%)	6.32*
Volunteering hours per week in the past 6 months at baseline	1.81 (2.94)	1.78 (2.47)	1.84 (3.37)	0.17
Volunteering intention at baseline	3.69 (1.06)	3.54 (1.12)	3.88 (0.95)	2.88**
Perceived volunteering opportunities during COVID-19^^^	4.28 (0.73)	4.33 (0.77)	4.22 (0.69)	1.08

*Notes*: COVID-19 = coronavirus disease 2019; *SD* = Standard Deviation; ^#^1 HKD = 0.13 USD. The percentages of employment status and self-rated health do not add up to 100% due to missing data. Missing rates for employment status, self-rated health, length of residence, types of voluntary work in the past 12 months, and perceived volunteering opportunities during COVID-19 were 2.6%, 0.9%, 4.3%, 2.6%, and 0.9%, respectively.

^^^Perceived volunteering opportunities during COVID-19 were measured by a single question asking participants to rate their perception of whether such opportunities decreased during the pandemic on a scale of 1 (strongly disagree) to 5 (strongly agree).

***p < .001. ** *p* < .01. * *p* < .05.

In addition, the timebank group had a higher volunteer participation rate in the past 6 months at baseline (65.5%) than the comparison group (59.1%) (*t* = 6.32, *p* < .05), although the comparison group had higher volunteer intention than the timebank group (*t* = 2.88, *p* < .01). No significant difference in weekly volunteer hours at baseline was found between the groups. Supplementary Table 3 and Supplementary Figure 1 show a detailed comparative analysis of outcome measures for both groups over time.

**Figure 2.**
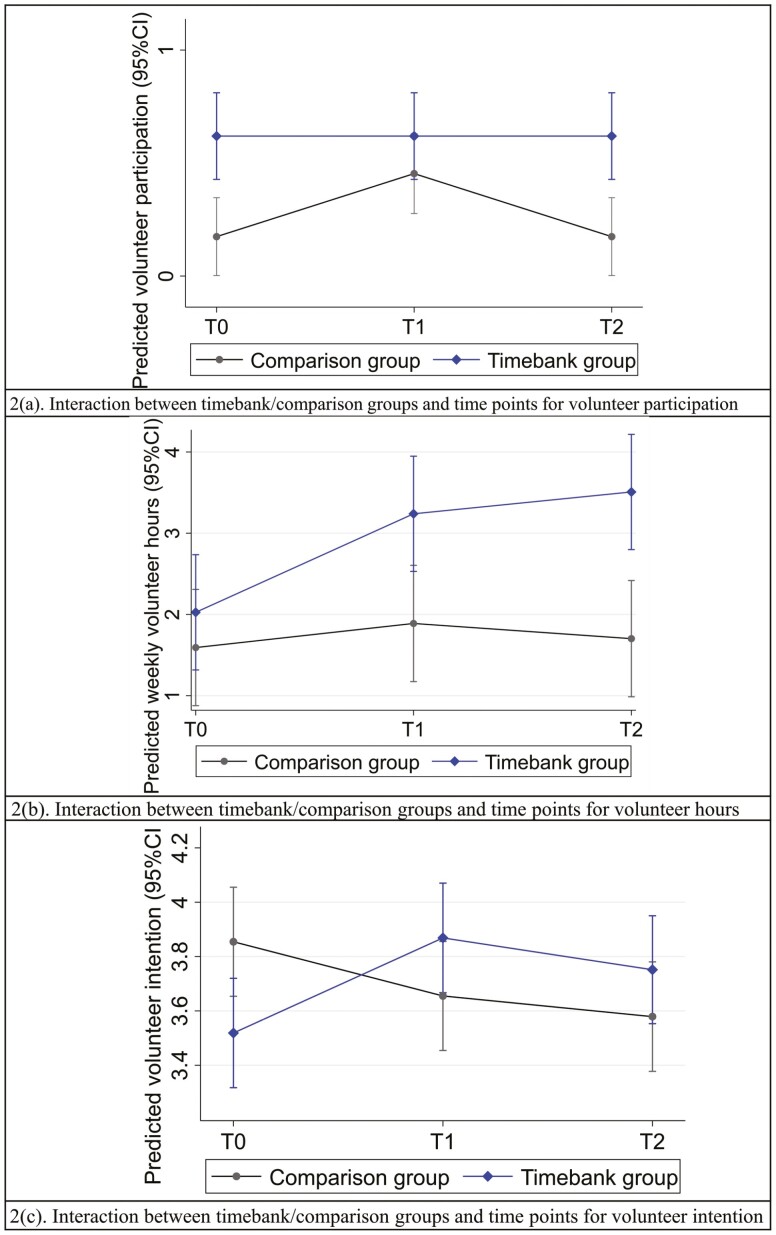
Interaction terms. CI = confidence interval.

Table 2 summarizes the results of mixed models. After controlling for covariates, the timebank group exhibited a significantly lower volunteer participation rate at T1 than the comparison group (interaction term between group condition and timepoints = −1.12, 95% CI: −2.18 to −0.05, *p* = .041), providing no evidence to support Hypothesis 1. Conversely, the timebank group showed significantly larger effects in increasing weekly volunteer hours (AMD = 1.37, 95% CI: 0.20–2.54, *p* = .021) at T2 than the comparison group, supporting Hypothesis 2. Meanwhile, the timebank group showed significantly larger effects in increasing the level of volunteer intention at T1 (AMD = 0.55, 95% CI: 0.25–0.87, *p* < .001) and T2 (AMD = 0.52, 95% CI: 0.20–0.83, *p* = .001), supporting Hypothesis 3. Figure 2A–C provides a visualization of the interaction effects of group conditions and time points: the timebank group maintained high volunteer participation rates, whereas the comparison group’s participation rates increased at T1 but returned to initial levels at T2; the timebank group exhibited an upward trend in weekly volunteer hours over time, while the comparison group sustained a low level; the timebank group displayed a substantial increase in volunteer intention between T0 and T1 with a slight decrease at T2, whereas the comparison group revealed a downward trend over time.

**Table 2.** Adjusted Difference in Volunteering Participation, Volunteering Hours, and Volunteering Intention Between Timebank and Comparison Groups Using Logistic/Linear Mixed Models (*N* = 230)

Variables	Volunteer participation			Weekly volunteer hours			Volunteer intention		
	β (95% CI)	*SE*	*p*	β (95% CI)	*SE*	*p*	β (95% CI)	*SE*	*p*
TBG vs CG	1.5 (0.5, 2.51)	0.51	.003	0.31 (−0.69, 1.32)	0.51	.542	−0.42 (−0.69, −0.14)	0.14	.003
Time point (ref: T0)									
T1	1.1 (0.38, 1.83)	0.37	.003	0.3 (−0.53, 1.13)	0.42	.474	−0.2 (−0.42, 0.02)	0.11	.076
T2	0 (−0.7, 0.7)	0.36	1.000	0.11 (−0.72, 0.94)	0.42	.796	−0.28 (−0.5, −0.06)	0.11	.013
Interaction (ref: T0)									
TBG × T1	**−1.1 (−2.17, −0.04)**	0.54	**.042**	0.91 (−0.26, 2.08)	0.6	.127	**0.54 (0.23, 0.86)**	**0.16**	**.001**
TBG × T2	0 (−1.04, 1.04)	0.53	1.000	**1.37 (0.2, 2.54)**	0.6	**.021**	**0.51 (0.2, 0.82)**	**0.16**	**.001**
*Covariates*									
Age	0.03 (−0.05, 0.1)	0.04	.443	0.05 (−0.02, 0.12)	0.04	.163	−0.01 (−0.03, 0.01)	0.01	.306
Married	−0.09 (−0.92, 0.73)	0.42	.823	0.66 (−0.14, 1.46)	0.41	.108	−0.09 (−0.32, 0.14)	0.12	.433
Education (ref.: primary or below)									
Secondary	0.3 (−1.06, 1.66)	0.69	.662	1.05 (−0.25, 2.34)	0.66	.113	0.38 (0.01, 0.74)	0.19	.044
Tertiary	−0.66 (−2.14, 0.82)	0.75	.381	0.15 (−1.27, 1.57)	0.72	.836	0.11 (−0.29, 0.51)	0.2	.584
Monthly salary (HK$) (ref.: below $2 000)									
$2 000–$5 999	0.61 (−0.5, 1.71)	0.56	.284	−0.11 (−1.19, 0.96)	0.55	.837	−0.08 (−0.38, 0.23)	0.15	.615
$6 000–$9 999	0.53 (−0.79, 1.85)	0.67	.434	0.42 (−0.86, 1.7)	0.65	.519	−0.09 (−0.46, 0.28)	0.19	.621
$10 000 and above	−0.75 (–1.68, 0.17)	0.47	.110	0.01 (−0.89, 0.92)	0.46	.978	−0.2 (−0.46, 0.06)	0.13	.129
Female	0.15 (−0.89, 1.19)	0.53	.783	0.85 (−0.17, 1.87)	0.52	.101	−0.17 (−0.46, 0.12)	0.15	.239
Not employed (ref.: employed)	0.1 (−1.19, 1.39)	0.66	.879	0.11 (−1.12, 1.35)	0.63	.855	−0.17 (−0.53, 0.19)	0.18	.360
Length of residence (years)	0 (−0.02, 0.02)	0.01	.765	−0.01 (−0.03, 0.01)	0.01	.412	0 (−0.01, 0)	0	.502
Length of volunteering (years)	0.03 (−0.01, 0.07)	0.02	.087	0.09 (0.05, 0.12)	0.02	<.001	0.01 (0, 0.02)	0.01	.012
Types of participation in voluntary work at baseline									
Social service-related	1.65 (0.81, 2.49)	0.43	<.001	1.09 (0.33, 1.85)	0.39	.005	0.26 (0.04, 0.48)	0.11	.020
Religious-related	1.31 (0.23, 2.4)	0.55	.018	−0.03 (−1.03, 0.97)	0.51	.955	0.02 (−0.26, 0.31)	0.14	.867
Regional organization	2 (0.84, 3.16)	0.59	.001	1.52 (0.46, 2.57)	0.54	.005	0.59 (0.29, 0.88)	0.15	<.001
Others	0.68 (−0.22, 1.57)	0.46	.137	0.34 (−0.51, 1.19)	0.43	.437	0.37 (0.13, 0.62)	0.12	.003
Self-rated good health	0.58 (−0.26, 1.42)	0.43	.179	−0.1 (−0.93, 0.73)	0.42	.808	0.19 (−0.04, 0.43)	0.12	.110
Volunteer opportunities during COVID-19	−0.09 (−0.62, 0.44)	0.27	.745	−0.4 (−0.9, 0.09)	0.25	.11	−0.03 (−0.17, 0.12)	0.07	.713
Constant	−8.93 (−15.48, −2.37)	3.34	.008	−5.61 (−11.75, 0.52)	3.13	.073	3.47 (1.71, 5.22)	0.89	<.001
ICC	0.56 (0.43, 0.69)			0.28 (0.2, 0.38)			0.31 (0.23, 0.4)		

*Notes*: CG = comparison group; β = coefficient; CI = confidence interval; COVID-19 = coronavirus disease 2019; ICC = intracluster correlation; TBG = timebank group; *SE* = standard error. ICC which measures the proportion of the overall variance in the outcome which is explained by within person variance compared with the time-level variance. A logistic mixed model with random intercepts was used for estimating the volunteering participation, and linear mixed models with random intercepts were applied for estimating weekly volunteering hours and volunteering intention. Covariates include age, sex, marital status, monthly income, education, employment status, length of residence and length of volunteering experience, types of voluntary work participation in the past 12 months, self-rated health, and the perceived volunteering opportunities during COVID-19.

In the timebank group, Supplementary Table 4 indicates that 25 (21.6%) participants kept the rewards for personal use, 18 (15.5%) shared rewards with family and friends, and a small number (6%) donated the rewards to strangers. Table 3 reveals that the timebank volunteers who retained rewards for personal use at least once significantly increased their weekly volunteer hours at T2 compared with those without such experience (β = 2.09, 95% CI: 0.43, 3.76, *p* = .014). However, neither sharing rewards with family and friends nor donating them to strangers significantly affected volunteer hours or intentions.

**Table 3.** Adjusted Difference in Volunteering hours and Volunteering Intention Across Subgroups of the Choice to use Rewards in the Timebank Group (*N* = 116)

Variables	Weekly volunteer hours			Volunteer intention		
	β (95% CI)	*SE*	*p*	β (95% CI)	*SE*	*p*
Frequency of reward sharing				Ref: CG		
Retaining the rewards for self-use (ref.: none)						
One and above	**2.09 (0.42, 3.76)**	0.85	**.014**	0.31 (−0.26, 0.87)	0.28	.286
Rewards sharing with social circles (ref.: none)						
One and above	−0.74 (−2.38, 0.89)	0.83	.372	−0.07 (−0.74, 0.59)	0.34	.826
Donating rewards to strangers (ref.: none)						
One and above	1.1 (−0.72, 2.93)	0.93	.236	−0.16 (−1.01, 0.69)	0.43	.716

*Notes*: β = coefficient; CI = confidence interval. GLM was applied for estimating the effects of types of relationship with people the rewards shared with on the weekly volunteering hours, while OLS linear regression was used for the volunteer intention. Covariates include age, sex, marital status, monthly income, education, employment status, length of residence and length of volunteering experience, types of voluntary work participation in the past 12 months, self-rated health, and the perceived volunteering opportunities during COVID-19. No findings for volunteer participation were generated from the logistic model due to the small numbers claiming rewards. The bold value indicate a statistically significant result.

### Qualitative Findings

The qualitative findings further explored participants’ perceptions of rewards, their experiences utilizing rewards, and the impact of rewards on their volunteering.

Regarding reward perception, we identified a theme of “*role recognition*.” All focus group participants from the timebank group reported that rewards served as recognition of their contributions. Regardless of whether they claimed rewards or not, they agreed that the rewards represented “respect,” “appreciation,” and “recognition” for their value to organizations, communities, and society (Supplementary Table 5, Q1–Q5).

We identified four themes explaining the effect of rewards in increasing volunteer behaviors and intentions. First, *interesting experiences generate a sense of meaningfulness when using rewards*. Participants acknowledged that they valued the rewards (e.g., a day trip or dining coupons) provided by social enterprises, and these rewards expanded and enriched their life experiences (Supplementary Table 5, Q6–Q7). This reward design motivated them to volunteer more in the future. Second, *a sense of fulfillment when sharing rewards with family and friends*. Participants reported feeling happy, satisfied, and fulfilled when sharing rewards (e.g., cookies offered by the social enterprises) with family and friends (Supplementary Table 5, Q8–Q9). This sentiment arose from generating positive spillover effects (e.g., bringing happiness to family and friends) through earned rewards. Lastly, participants reported *the reinforcement of prosociality when sharing rewards with their friends,* as this provided an opportunity to share stories and volunteering experiences behind the rewards. Hence, they perceived themselves as role models in their social circles, using their influence to attract and motivate friends to volunteer, thus reinforcing their motivation to volunteer more (Supplementary Table 5, Q10).

We identified three themes that explained not claiming rewards. First, participants who did not claim rewards emphasized *safeguarding their intrinsic motivation for volunteering*, which was not driven by a desire for rewards (Supplementary Table 5, Q11–Q12). This principle should be maintained even in the presence of rewards. Second, participants intending to claim rewards did not do so to *avoid appearing greedy*; simple acts such as asking social workers how to claim the rewards made them feel greedy (Supplementary Table 5, Q13–Q14). Lastly, participants who might otherwise have claimed the rewards commented on the *digital barriers* to using the Reward redemption function in HINCare (Supplementary Table 5, Q15–Q16).

## Discussion

To the best of our knowledge, this is the first study to test the effectiveness of timebanking on promoting volunteer behaviors and intention among older adults using a quasi-experimental mixed-method design. The study showed that timebanking encouraged older adults to volunteer more and increased volunteer intention. Additional insights were generated from this study.

This study indicates that timebanking does not effectively enhance volunteer participation compared with the nonreward condition; thus Hypothesis 1.1 was not supported. Further analysis showed that the timebank group sustained a high volunteer participation rate over time, and participants were drawn to the HA program from other voluntary activities (Supplementary Table 3). Volunteer participation within the timebank group may already have been high, potentially leading to ceiling effects on volunteer participation. An alternative interpretation is that prompting individuals who have never previously volunteered (whether due to lack of interest or other reasons) to start volunteering, especially in the context of COVID-19 (Sun et al., 2021) may be challenging. Nevertheless, our study suggests that timebanking attracted older adults, outperforming other volunteer programs, implying that although timebanking may not significantly increase the overall rate of volunteer participation, it could help sustain a high level of participation.

More importantly, our findings support Hypotheses 1.2–1.3, showing that timebanking increases volunteer hours and intentions compared with nonreward conditions, aligned with social exchange theory (Cook et al., 2013). Our qualitative findings revealed that the timebank group felt recognized for their contributions, which may balance the costs and benefits of volunteering, leading to increased volunteer engagement and future intentions (Cahn & Gray, 2021). These findings resonate with Tse (2020) findings that volunteers who felt greater respect from others were more likely to continue volunteering. In contrast, those with lower levels of perceived respect were more likely to withdraw over time. Our findings highlight the importance of rewards in motivating people to sustain volunteering. Volunteering should not be viewed merely as a unidirectional “giving” activity but rather as a reciprocal exchange process (Zaninotto et al., 2013). Timebanking reinforces this reciprocal exchange process through a time credit-reward mechanism, allowing older volunteers to feel recognized and respected, and encouraging them to volunteer more.

Furthermore, this study is the first to deliver nuanced evidence regarding how reward sharing affects subsequent volunteerism, thus expanding existing literature on late-life volunteerism focusing on how feelings of being respected or recognized influenced late-life volunteering (Tse, 2020). The quasi-experimental study findings indicate that those who used the rewards for themselves volunteered more at T2 than those without such experience. Our qualitative study provided a deeper understanding by revealing that participants derived a sense of meaningfulness from their experiences of using rewards, appreciating the rewards offered by social enterprises, and noting how these enhanced their life experiences. These insights underscore the potential of rewards not only as a means of attracting volunteers but also as a tool to retain them and enhance their volunteering experience.

Interestingly, although our quantitative study did not provide evidence of the impact of sharing rewards with friends and family on subsequent volunteerism, our qualitative study revealed that participants who shared rewards within their social circle experienced a sense of fulfillment and a reinforcement of their volunteer intention, generating positive spillover effects, such as motivating their friends to volunteer. The benefits realized from sharing rewards with close relations could potentially amplify the perceived advantages of volunteering. These findings align with inclusive fitness theory (Hamilton, 1964) and socioemotional selectivity theory (Carstensen, 2021). The former proposes that humans are more prosocial towards individuals they feel socially close to, rather than strangers, to optimize the survival rate of their own genes. This can also apply to sharing rewards with friends if such actions indirectly enhance an individual’s overall fitness through improved social bonds and reciprocal support. The latter suggests that, as they age, individuals prioritize relationships with close social partners to fulfill emotionally meaningful goals and sharing rewards within their social circle enhances the positive experiences associated with social engagement. These theories provide a biological and psychological basis for understanding how reward sharing with close social connections might foster volunteerism in later life. The divergence observed between the quantitative and qualitative outcomes concerning reward sharing within social circles may be attributed to only a minority of participants (15.5%) intending to share rewards with close social connections. Future studies could investigate how various reward-sharing behaviors—such as retaining rewards for personal use or sharing with family and relatives, friends, or strangers—influence volunteer motivation and intention. Such research could illuminate the nuanced ways in which rewards could be distributed to people with different social relationships with volunteers and affect the dynamics of volunteering. This could lead to developing more effective strategies for sustaining volunteer activities.

Finally, our qualitative findings identify several reasons why timebank group participants chose not to claim rewards. Notably, preserving the intrinsic motivation for volunteering was a primary consideration. This is consistent with previous research asserting that motivations for volunteering extend beyond mere reward (Morrow-Howell, 2010). A prominent intrinsic motivator for volunteering later in life is generativity—a desire to make a societal contribution (Yamashita et al., 2019). Our qualitative study does not suggest that the availability of rewards negates the intrinsic motivation to volunteer. Instead, it stresses the importance of recognizing and supporting the motivations that encourage older adults to volunteer. It also highlights the need for a careful approach to reward systems within timebanks, ensuring that they do not undermine the intrinsic motivation of volunteering, although still offering some forms of acknowledgment that may encourage participation. In addition, we identified obstacles in the reward claiming process, such as issues related to information access or technical complications, which could hinder older volunteers from claiming their rewards (O’Connell et al., 2022). These insights underscore the importance of ensuring that the reward systems are user-friendly and accessible, particularly for older adults who may face technical or information barriers.

## Strengths and Limitations

Our study has several strengths. First, it is pioneering research to employ a quasi-experimental study design to establish the causality between timebanking and late-life volunteering. This design minimizes contamination risks and offers robust, high-quality evidence supporting the effectiveness of timebanking on promoting late-life volunteerism, as many existing timebanking studies are qualitative or case studies (Lee et al., 2020). Second, we break new ground by innovatively examining how reward-sharing influences volunteerism in later life. Last, we used the qualitative study to expand on the quasi-experimental study’s findings, offering a rich, in-depth understanding of participants’ viewpoints on rewards and their experiences utilizing them.

Nevertheless, our study has several limitations. First, our methodology did not include random assignment of participants into the timebank and comparison groups. The two groups significantly differed in age, volunteer experience, and volunteer participation rates, although we controlled these covariates to estimate the adjusted difference in volunteerism. Meanwhile, our study drew on a nonrepresentative sample, which should caution against broad generalizations. Our sample was also small, and thus the study may not have been sufficiently powered to detect a difference in changes in outcomes between the groups. Second, this quasi-experimental study design did not consider the types or forms of rewards. Volunteers’ motivations might be influenced by their preferences for various rewards, an aspect we did not explore. Third, due to COVID-19 restrictions, rewards became available 6 months after the timebank program’s announcement in reward districts, limiting the time available for the timebank group to exchange their rewards. The effects of COVID-19 also diminished opportunities, intentions, and actual participation time for both groups, resulting in a significant dropout rate (e.g., 30%). Although we controlled for the perceived volunteer opportunities during COVID-19, the effects of timebanking on promoting late-life volunteering might be underestimated. Fourth, several participants in the qualitative study mentioned barriers to using the HINCare App, leading to a potential underestimation of the timebank’s effect. We did not include participants’ ability to use technology in the covariates. Fifth, the outcome measurements included the self-reported participation and hours of volunteering, subject to recall bias and social desirability bias. Lastly, our study focused on the short-term effects (e.g., 1 year) of timebanking on promoting late-life volunteering. The long-term effects of such an initiative warrant further investigation.

## Future Research and Implications

Several important questions remain unanswered, including the mechanisms through which timebanking promotes late-life volunteering and whether the effects of timebanking vary according to volunteers’ levels of intrinsic motivation. Our qualitative research identified potential mechanisms of timebanking, such as a sense of recognition, perceived meaningfulness, a sense of fulfillment, and reinforcement of prosociality. Future research could further investigate the operation of these potential mechanisms. Furthermore, our qualitative study revealed that some volunteers were reluctant to claim rewards so as not to undermine their intrinsic motivation for volunteering, thus raising the question of whether the effects of timebanking on late-life volunteering might differ among individuals with varying levels of intrinsic motivation. This needs further research. Understanding the interplay between extrinsic rewards and intrinsic motivations could be pivotal in designing and implementing effective timebanking systems that promote sustained and meaningful volunteer engagement, particularly among older adults. It is also noteworthy that implementing timebanking for volunteer rewards entails both administrative and technological expenses. Future research could evaluate the cost-effectiveness of timebanking, particularly in fostering widespread volunteerism among older adults.

This study offers critical insights for practitioners and policymakers, especially with increasing interest in promoting volunteering among older adults, such as through social prescribing. Our results can be leveraged to create reward-based timebanking platforms to encourage widespread mutual aid in societies with aging populations that could enhance the visibility of older adults’ contributions to society by acknowledging their volunteer efforts. Second, the positive outcomes of rewarding volunteers, their families, and friends can be a powerful incentive for future volunteering. When older adults share their rewards, it creates a ripple effect of benefits. Policymakers could underscore these positive spillover effects within social circles, helping potential volunteers grasp the broader impact of their reward utilization behaviors. Finally, as integrating technology into volunteering gains traction (Perez-Vega & Miguel, 2022), there should be an emphasis on encouraging technical innovations that streamline service exchanges and rewards for older volunteers. This will optimize the benefits of timebanking in promoting volunteering late in life (Chui & Chan, 2019).

## Conclusion

This study substantiates that timebanking, a reward-based system, effectively enhances volunteerism among older adults and bolsters intentions for sustained volunteering. Our qualitative study revealed that recognition is a potential lever in timebanking’s influence. Using rewards personally enriched the volunteer experience, and those sharing rewards with their family and friends experienced fulfillment. Policymakers should recognize timebanking as a social innovation capable of providing a supportive platform that empowers older adults to contribute to society meaningfully. Given a dwindling workforce and escalating demands for long-term care, the urgency for such social innovations is compelling.

## Supplementary Material

igae056_suppl_Supplementary_Materials

## Data Availability

Data and materials are not available because authors have not completed their original work with the data set. The study reported in the manuscript was not preregistered. However, We registered this study with the Centre for Open Science (https://osf.io/dmfvh).
