# Supplementary material for: Promoting Late-Life Volunteering With Timebanking: A Quasi-Experimental Mixed-Methods Study in Hong Kong
Source: Innov Aging. 2024 Jun 4;8(7):igae056. doi: 10.1093/geroni/igae056 (PMC11275466; doi:10.1093/geroni/igae056)
Supplement: igae056_suppl_Supplementary_Materials [file igae056_suppl_supplementary_materials.docx]

***Innovation in Aging* Supplementary Material: Lu et al. Promoting Late-Life Volunteering with Timebanking: A Quasi-Experimental Mixed-Methods Study in Hong Kong.**

Supplementary Table 1. Comparisons between timebank and comparison districts

| Variables | Intervention districts | comparison districts | t-test |
| --- | --- | --- | --- |
| Percentage of people aged 65 and above | 0.23 (0.01) | 0.21 (0.02) | 2.121 |
| Percentage of people completing post-secondary and higher education | 0.34 (0.07) | 0.44 (0.15) | -1.055 |
| Workforce participation rate | 58.47 (1.89) | 61.9 (4.81) | -1.151 |
| Median monthly income (HK$) | 19000 (1732.05) | 25500 (7794.23) | -1.410 |

Notes: SE = standard error. No significant difference in sociodemographics between intervention and comparison districts were found.

Supplementary Table 2. Characteristics of focus groups participants (N = 17)

| Participant  ID code | Sex | Age | Subgroup |
| --- | --- | --- | --- |
| 1109EA | Female | 71 | Did not redeem rewards |
| 1109EB | Male | 70 | Did not redeem rewards |
| 1109EC | Female | 80 | Did not redeem rewards |
| 1109ED | Female | 67 | Did not redeem rewards |
| 1109EE | Female | 73 | Did not redeem rewards |
| 1109EF | Female | 56 | Did not redeem rewards |
| 1111E1 | Female | 76 | Redeemed rewards |
| 1111E2 | Male | 66 | Redeemed rewards |
| 1111E3 | Male | 79 | Redeemed rewards |
| 1111E4 | Female | 72 | Redeemed rewards |
| 1111E5 | Female | 66 | Redeemed rewards |
| 1114E1 | Female | 65 | Did not redeem rewards |
| 1114E2 | Female | 58 | Did not redeem rewards |
| 1114E3 | Female | 68 | Did not redeem rewards |
| 1114E4 | Male | 70 | Did not redeem rewards |
| 1114E5 | Female | 64 | Did not redeem rewards |
| 1114E6 | Male | 65 | Did not redeem rewards |

Supplementary Table 3. The unadjusted difference in volunteering participation, volunteering hours and volunteering intention between timebank and comparison groups (N=230)

| Variables | Timebank group | Comparison group | Comparison (TBG-CG) | |
| --- | --- | --- | --- | --- |
|  |  |  | Unadjusted Diff | Chi-square/ t-test |
| Volunteering participation (%) |  |  |  |  |
| T0 | 65.50% | 49.12% | - | 6.32* |
| T1 | 65.50% | 64.04% | - |  |
| T2 | 65.50% | 49.12% | - |  |
| *Chi-square* | 0 | 6.81* |  |  |
| Beyond HA program volunteering participation (%) |  |  |  |  |
| T0 | 65.52% | 49.12% | - | 6.32* |
| T1 | 56.90% | 59.65% | - |  |
| T2 | 50.86% | 48.25% | - |  |
| *Chi-square* | 5.16 | 3.68 |  |  |
| HA program volunteering participation (%) |  |  |  |  |
| T1 | 37.07% | 6.14% | - | 32.33*** |
| T2 | 45.69% | 9.65% | - | 37.12*** |
| *Chi-square* | 1.78 | 0.96 |  |  |
| Volunteering hours per week (Mean, SD) | |  |  |  |
| T0 | 1.78 (2.47) | 1.84 (3.37) | -0.06 | -0.17 |
| T1 | 2.99 (4.02) | 2.15 (3.14) |  |  |
| T2 | 3.26 (6.66) | 1.95 (3.27) |  |  |
| ANOVA | 3.26* | 0.25 |  |  |
| Volunteering intention (Mean, SD) | |  |  |  |
| T0 | 3.54 (1.12) | 3.88 (0.95) | -0.40 | -2.88** |
| T1 | 3.86 (1.19) | 3.68 (1.06) |  |  |
| T2 | 3.78 (1.14) | 3.63 (1.13) |  |  |
| ANOVA | 4.82** | 2.41 |  |  |

Notes: SD=Standard Deviation; *** p < 0.001, ** p < 0.01, * p < 0.05

Supplementary Table 4. The frequencies of reward sharing behaviors within the timebank group (N=116)

| Variables | N (%) |
| --- | --- |
| Retaining the rewards for self-use |  |
| Once and above | 25 (21.6%) |
| None | 91 (78.4%) |
| Rewards sharing within social circle |  |
| Once and above | 18 (15.5%) |
| None | 98 (84.5%) |
| Reward sharing with strangers |  |
| Once and above | 7 (6%) |
| None | 119 (94%) |

| **The meaning of rewards in the timebanking system** | |
| --- | --- |
| #1 | *After receiving rewards, it means recognizing my contribution. Also, it recognizes how long I have been volunteering in the community. In other words, it (volunteer organization) does not neglect volunteers’ [contributions] [1109EB]* |
| #2 | *It is better to get recognition [via receiving rewards] than nothing. [Getting rewards] is recognition and encouragement to [volunteer]. [1109ED]* |
| #3 | *It’s just like a recognition for helping and getting things done. It has nothing to do with the value of [rewards], it just makes you feel happy when you have done something and been recognized by others*. [*1114E5]* |
| #4 | *[Offering rewards] represents our contribution has been respected, it [rewards] is something extra for us. [1111E4]* |
| #5 | *It means you have done something and someone respected [your contribution], and so giving back some compensation to you. [1111E1]* |
| **Reasons why rewards may boost volunteering hours or intention** | |
| #6 | *My most appreciated reward is the Lai Chi Woo day trip, it is really good and meaningful experience…very worth to go…as [I] never been there before. [1111E1]* |
| #7 | *Using my time credits to exchange for dining coupons and trying out the restaurants run by social enterprises in Wanchai was a novel and enjoyable experience. [1111E4]* |
| #8 | *I shared [cookies] with my relatives and friends. After receiving cookies, they [friends] thanked me. 1111E2* |
| #9 | *I invited ten people to go to [Lai Chi Woo day trip]. Many people asked me to invite them and so I invited my family and some volunteer friends…[I am] so happy. [1111E1]* |
| #10 | *About the cookies…when I went hiking, I shared them with my hiking buddies, [I] opened the [cookies], and gave one to each of them. Then, someone [hiking buddies] asked where I got the cookies. I replied that I got them from the timebanking program. Since I gained some credits via volunteering that can be redeemed. I used credits to redeem cookies. So, we enjoyed the cookies and chatted. I also tried to use cookies to attract them to volunteer. Four of them have started volunteering. [1111E3]* |
| ***Reasons volunteers did not redeem rewards.*** | |
| #11 | *[I] did not need to receive credits for what I have done. Personally, volunteers do not need to have rewards. To be honest, personally, I think the rewards are useless for me. As I genuinely want to volunteer, I don’t care if no one compliments me. [1109EA]* |
| #12 | *I think redeeming rewards is so troublesome therefore I did not redeem them. [1114E2]* |
| #13 | *I really need it [redeeming rewards], but it is too troublesome so that I do not need it. I have no point to accumulate. [1109EF]* |
| #14 | *Regarding the timebank, the timebanking project has sent the HINCare account to me, but the staff from the volunteer organization center were so busy. Sometimes, I did not how to use the [HINCare App] and asked them about it. They told me to wait again and again so I cancelled it[HINCare App]. It is useless for me as I never aim for these [rewards to volunteer]. [1109EB]* |
| #15 | *I think [HINCare App] is not a user-friendly App. [1109EF]* |
| #16 | *[I think] it is so complicated to [use the HINCare App.[1109ED]* |

Supplementary Table 5. Qualitative findings

Notes: The numbers in brackets correspond to participant ID codes, which are connected to the descriptive information provided in Supplementary Table 2.

Supplementary Figure 1. Graphs of the unadjusted difference in volunteer behaviors and intention between the Timebank and the comparison groups.

| **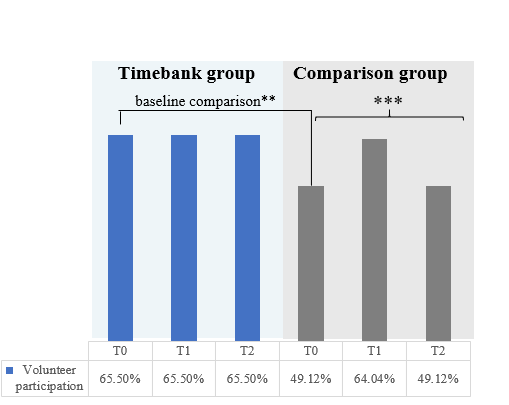** |
| --- |
| 1. **Volunteer participation** |
| **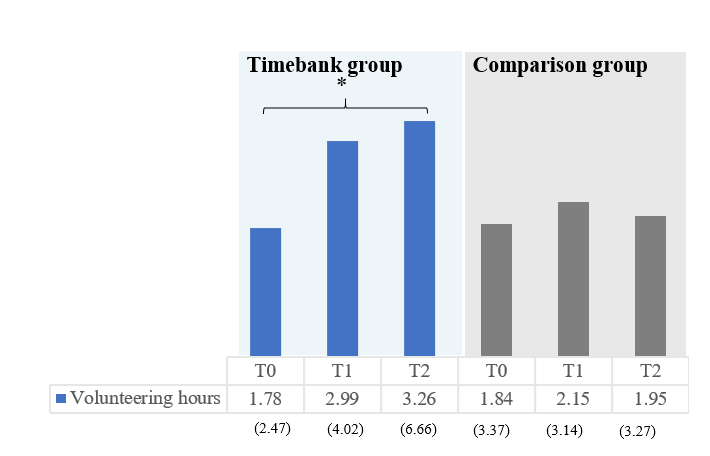** |
| 1. **Weekly volunteer hour** |
| 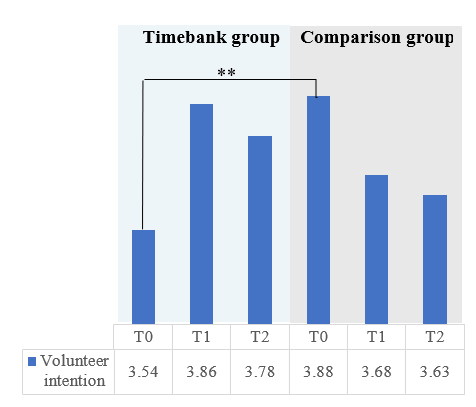 |
| 1. **Volunteer intention** |

Notes: Chi-square tests were used to test the unadjusted difference in the volunteer participation at baseline, in HA participation at during- and post-timebank. Independent samples T-test was used to test the unadjusted difference in the weekly volunteer hours and volunteer intention between two groups. *** p < 0.001, ** p < 0.01, * p < 0.05
